# Mild phenotypes associated with an unbalanced X‐autosome translocation, 46,X,der(X)t(X;8)(q28;q13)

**DOI:** 10.1002/ccr3.1596

**Published:** 2018-06-24

**Authors:** Takafumi Watanabe, Makiho Ishibashi, Ryota Suganuma, Miki Ohara, Shu Soeda, Hiromi Komiya, Keiya Fujimori

**Affiliations:** ^1^ Department of Obstetrics and Gynecology Fukushima Medical University School of Medicine Fukushima Japan

**Keywords:** 8q13 trisomy, unbalanced translocation, X inactivation, X‐autosome, Xq28 monosomy

## Abstract

Unbalanced X‐autosome translocation can result in various phenotypic manifestations. We present the first case of 46,X,der(X)t(X;8)(q28;q13) in a 34‐year‐old female with relatively mild manifestations, including congenital heart disease, epicanthal fold, mild intellectual disability, and menstrual irregularity. Our findings expand the known spectrum of unbalanced X‐autosome translocations, for improved clinical management.

## 1 INTRODUCTION

Autosomal translocations are relatively common, with a frequency of about 1:500. However, X‐autosome translocations are very rare, occurring in about 1:30 000 live births.^1^ In females with X‐autosome translocations, the main causal factor of clinical phenotype is dependent on the X chromosome inactivation pattern.^2^ Two forms of translocations have been observed, balanced, and unbalanced, and various phenotypes were reported from normal to multiple congenital anomalies and intellectual disability. In the majority of cases with balanced X‐autosome translocation, there is preferential inactivation of the normal X chromosome because the inactivation of the derivative X chromosome causes an imbalance of gene expression, such as autosomal partial monosomy and partial disomy X and is frequently associated with abnormal phenotypes.^3^ Although females with balanced X‐autosome translocations are generally described as having a normal phenotype, ovarian dysfunction is observed when breakpoints are located between Xq13 and Xq26, which has been defined as the critical region.^4^, ^5^ On the other hand, in females with unbalanced X‐autosome translocations, the abnormal X chromosome is generally inactive because the inactivation of the normal X chromosome would result in a severe genomic imbalance. Their phenotypes are usually abnormal with characteristics such as multiple congenital anomalies and intellectual disability, due to partial aneuploidy of the X chromosome and the autosome. We herein describe, to our knowledge, the first case of an unbalanced X‐autosome translocation resulting in the deletion of Xq28‐qter and autosomal trisomy 8q13‐qter.

## 2 CLNICAL REPORT

A 34‐year‐old female, gravida 0, para 0, with about 5 years of infertility was referred to our hospital with her husband. Her menstruation was irregular, and she had had a radical operation for double outlet right ventricle when she was 6 years old. On examination, her height was 154 cm, weight was 52 kg, and she had an epicanthal fold and a mild intellectual disability. Her external and internal genitalia were normal. Hormonal studies indicated the following values: follicle stimulating hormone level, 17.69 mIU/mL (normal reference range in reproductive‐age women, 3‐20 mIU/mL); luteinizing hormone level, 7.21 mIU/mL (normal reference range in reproductive‐age women, 5‐20 mIU/mL); and estradiol level, 11.9 pg/mL (normal reference range in reproductive‐age women, 20‐400 pg/mL). Her prolactin level was normal. Her parents and older sister were mentally and physically normal.

**Figure 1:**
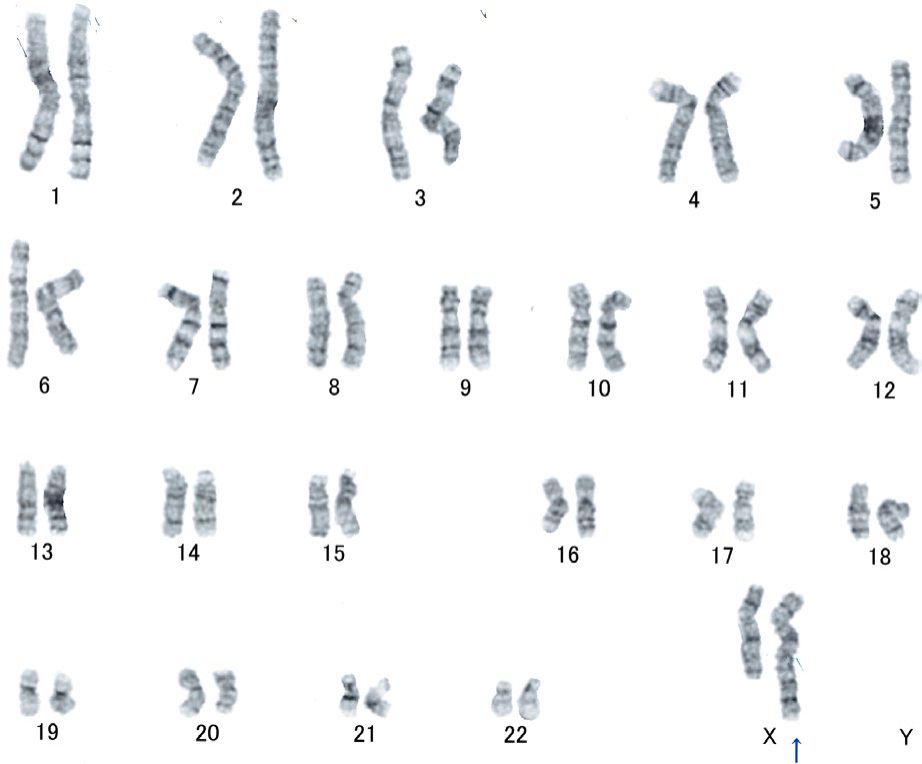
G‐banding karyotype of the patient. The arrow indicates the derivative X chromosome

**Figure 2:**
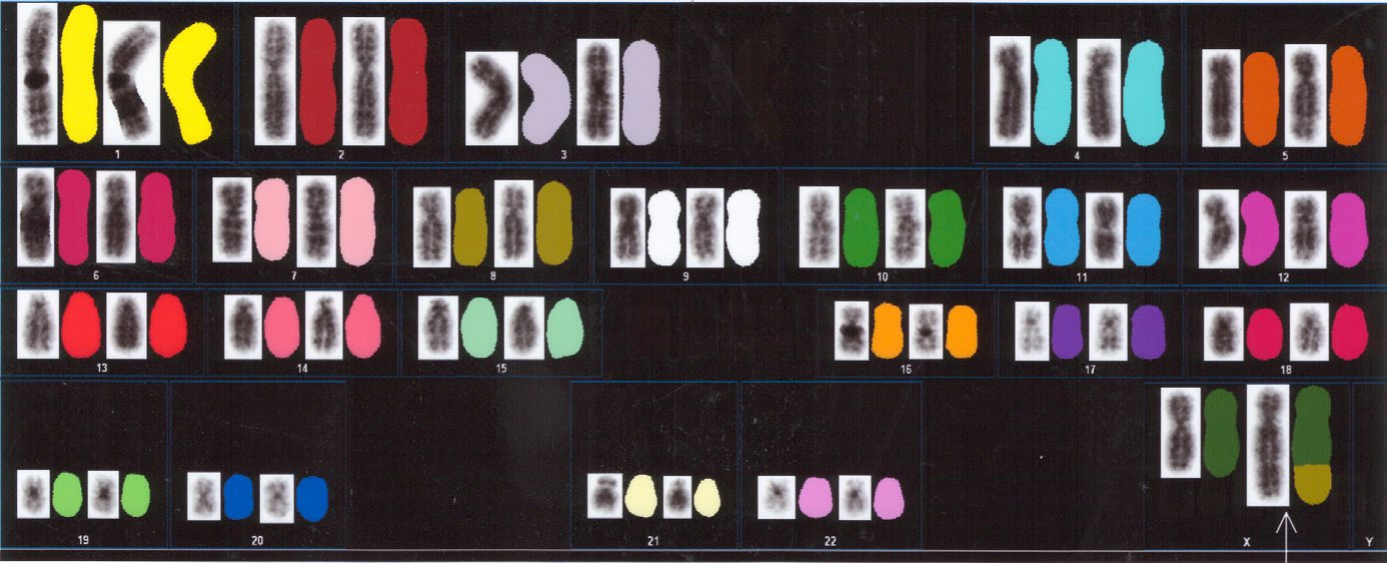
Spectral karyotyping analysis of the patient. Each chromosome is represented twice, by reverse DAPI staining on the left and by a spectral karyotyping analysis image shown in classification colors on the right. The arrow indicates the derivative X chromosome from t(X;8)(q28;q13)

Written informed consent was obtained, and genetic counseling was performed. A karyotype with a derivative X chromosome containing additional genetic material on its long arm was observed using the GTG‐banding technique (Figure 1). Spectral karyotyping analysis was performed to delineate the origin of the additional X chromosomal material. This analysis was conducted because the patient’s parents refused a cytogenetic examination. Spectral karyotyping analysis indicated that the additional chromosomal material was derived from the distal long arm of chromosome 8 with breakpoints at Xq28 and 8q13 (Figure 2). The complete karyotype was therefore identified as 46,X,der(X)t(X;8)(q28;q13). An unbalanced X‐autosome translocation resulted in the deletion of Xq28‐qter and trisomy for 8q13‐qter. After 4 cycles of Kaufmann therapy, ovulation induction using a follicle stimulating hormone and human chorionic gonadotropin was performed for World Health Organization group 3 anovulation. At the time of writing, although ovulation was confirmed, the patient is not yet pregnant.

## 3 DISCUSSION

In a previous study, cytogenetic and molecular analysis of premature ovarian failure (POF) in women carrying balanced X‐autosome translocations and X chromosome deletions identified a region on the long arm of the X chromosome from Xq13 to Xq26 that is critical for ovarian development and function.^6^ This region can be split into two functionally different portions: from Xq23 to q27 (POF1) and from Xq13 to q21 (POF2).^5^, ^7^, ^8^ The POF1 region has been associated with interstitial deletions, while the POF2 region has been interested by most of the breakpoints of balanced translocations.^7^ In females with Xq monosomy or Xq deletions, the risk of germ cell atresia is elevated due to haploinsufficiency of genes on the long arm of the X chromosome.^9^ Deletions of the long arm of the X chromosome result in POF and manifest as primary or secondary amenorrhea. POF‐associated genes have been identified on Xq critical regions.^10^ Although there is no clear demarcation of the discrete regions, it is useful to classify terminal deletions in the regions: Xq13‐q21, Xq22‐q25, and Xq26‐q28.^9^, ^11^ The partial monosomy arising from the Xq13‐21 regions is associated with primary amenorrhea and complete ovarian failure. Conversely, the partial monosomy occurring in the Xq22‐q25 regions is considered as POF rather than complete ovarian failure. The partial monosomy originating at the Xq26‐q28 regions has less effect on ovarian dysfunction. The patient in the present case had only menstrual irregularity, and her ovarian dysfunction did not appear to be severe. This mild ovarian dysfunction was considered to be due to the fact that the monosomy of Xq was a very small region at Xq28‐qter.

Complete trisomy 8 is usually an early lethal condition and can be a cause of miscarriage. Trisomy 8 mosaicism (Warkany syndrome) has an estimated frequency range of 1:25 000 to 1:50 000 births and displays extremely variable phenotypes ranging from normal to severe malformations.^12^ Pure distal 8q trisomy is a rare disorder and includes intellectual disability, growth impairment, dysmorphic facial features (prominent forehead, down‐slanting palpebral fissures, hypertelorism, a depressed nasal bridge, low‐set malformed ears, a long philtrum, micrognathia, and cleft palate), congenital heart disease, and urogenital anomalies.^13^, ^14^ Our patient had a relatively mild clinical phenotype including congenital heart disease (double outlet right ventricle), epicanthal fold, and mild intellectual disability, in spite of the large trisomic 8q13‐qter region. Although clinical phenotype severity is directly related to the size of chromosomal deletion or duplication, an unbalanced autosomal translocation can generally cause intellectual disabilities and physical abnormalities, or miscarriage. On the other hand, in cases of unbalanced X‐autosome translocations, inactivation of the derivative X chromosome can spread to the attached autosomal fragment, partially or fully, beyond the X chromosome.^15^ Some patients with unbalanced X‐autosome translocations manifest only mild behavioral problems because the gene expression of the partial trisomic regions disappears.^16^

In conclusion, the current case advances the understanding of X‐autosome translocation by presenting clinical and cytogenetic data regarding a new unbalanced chromosome X‐8 translocation. Although the X inactivation pattern was not confirmed using a molecular cytogenetic assay, we suspect that our patient has mild phenotypes because of the inactivation of the abnormal X chromosome 8q13‐qter and the presence of a small deletion, such as that of Xq28‐qter. To our knowledge, this is the first description of Xq28‐qter deletion with trisomy for 8q13‐qter. If the patient in the present study becomes pregnant, prenatal diagnosis using genetic amniocentesis with appropriate genetic counseling should be taken into consideration.

## CONFLICT OF INTEREST

None declared.

## AUTHORS’ CONTRIBUTIONS

TW: performed genetic counseling and drafted the manuscript. MI, RS, MO, SS, HC, and KF: performed the clinical assessment and approved the manuscript.
